# Carbon Nanostructure-Based DNA Sensor Used for Quickly Detecting Breast Cancer-Associated Genes

**DOI:** 10.1186/s11671-022-03730-3

**Published:** 2022-09-20

**Authors:** Yingqi Zhang, Jisu Song, Songlin Yang, Jianying Ouyang, Jin Zhang

**Affiliations:** 1 Chemical and Biochemical Engineering, University of Western Ontario, London, ON N6A 5B9 Canada; 2 School of Biomedical Engineering, University of Western Ontario, London, ON N6A 5B9 Canada; 3 National Research Council Canada, 1200 Montreal Road, Ottawa, ON K1A 0R6 Canada

**Keywords:** Carbon quantum dots, Graphene oxide, DNA sensor, Förster resonance energy transfer (FRET) quenching

## Abstract

The early diagnosis of breast cancer highly relies on the detection of mutant DNA at low concentrations. Förster resonance energy transfer (FRET) quenching may offer a solution to quickly detect a small amount of single-strand DNA (ssDNA) through the combination of nanomaterials with special luminescence and unique structures of DNA double helix structure. Here, carbon quantum dots (CDs) modified with Capture ssDNA act as the FRET donor which interact with the two-dimensional fluorescence quencher, i.e., graphene oxide nanosheets (GO), to detect breast cancer-associated Target ssDNA at a low concentration. CDs bioconjugated with the designed Capture ssDNA (named CDs-Capture ssDNA) have the maximum fluorescence intensity (*I*_max_) at the emission (*λ*_em_) = 510 nm. The fluorescence of CDs-Capture ssDNA is quenched, while they interact with GO due to the *π*–π* interaction between ssDNA and GO. In the presence of Target ssDNA, the *I*_max_ is restored because of the stronger interaction between Target ssDNA and CDs-Capture ssDNA through the hydrogen bond. The restored fluorescence intensity of CDs has a linear relationship with the concentration of Target ssDNA from 0.25 to 2.5 μM with a detection limit around 0.24 μM. The selectivity of the sensing system has been further evaluated by testing the 3-base mismatched and non-base matched in which efficient restoration of photoluminescence of the sensing system cannot be observed. This carbon nanostructure-based DNA sensing system offers a user-friendly and quick detection of single-strand DNA at lower concentration.

**Supplementary Information:**

The online version contains supplementary material available at 10.1186/s11671-022-03730-3.

## Introduction

DNA biosensors have been of interest for decades as researchers searched for rapid and accurate detection of sequence-specific DNA. Not only does DNA dictate the phenotype of a particular trait, but detection of specific mutated DNA also allows for early and accurate diagnosis of certain diseases, such as different types of cancers [1]. Fluorescent biosensors have shown to have great potential because of their rapid processing [2, 3]. Förster resonance energy transfer (FRET) technology, where a donor fluorophore transfers energy to an acceptor fluorophore given that they are within close proximity (< 10 nm), has been employed in detecting biomolecules by converting the biomolecular-involved process to measurable fluorescence properties [4]. The fluorescent transducer in FRET biosensor has been evolved from organic dyes to fluorescent nanostructures. Compared to organic dye which are sensitive to pH and temperature, and intend to have high photobleaching rate, nanostructures with fluorescence properties are normally considered better candidate as the fluorescent transducer because they have shown excellent properties, in terms of high photostability and with tunable excitation by adjusting the size of fluorescent nanostructures [5, 6].

Carbon-based nanomaterials are known to be environmentally friendly and biologically friendly materials, which allows them to be utilized in biosensing applications [7, 8]. In particular, carbon quantum dots (CDs) are of interest due to their strong fluorescence intensity, excitation-dependent emission, and good photostability; they have been found to be a superior alternative for traditional fluorophores. Furthermore, in comparison with semiconductor quantum dots (QDs), CDs are better alternatives as they display superior biocompatibility resulting in minimal cytotoxic effects [9]. In addition, CDs show lower cytotoxicity which can be applied in bioimaging [10]. In addition, the multicolor CDs have been studied for identifying biomolecules [11].

The energy transfer efficiency (*E*, i.e., the fraction of energy transferred) is reverse proportional to the distance of two fluorophores as shown in Eq. 1; [4]

1 $$E = \frac{1}{{\left[ {1 + \left( {\frac{r}{{R_{o} }}} \right)^{6} } \right]}}$$

where *r* is the distance between two fluorophores, *R*_0_ the distance at which 50% *E* was achieved. *R*_0_ is a characteristic parameter for given partners at given medium. Therefore, in design of traditional FRET pairs where the donor transfers energy to the acceptor leading to emission of photon at a longer wavelength, additional requirement in suitable excitation and emission of the donor and acceptor should be considered, whereas a FRET quenching system just requires that the acceptor has an absorbance at the wavelength overlapping with the emission of donor. In addition, the fluorescence intensity normally decreases with increasing concentration of the targeted molecules in traditional FRET biosensor, whereas the fluorescence intensity increases with an increase in the concentration of the targeted molecules in FRET quenching biosensing system. Therefore, biosensors by applying FRET quenching system show an advantage over FRET system in detection of small amount of analytes [12, 13]. By using organic quenching molecules, it has been applied in studying the protein–protein interaction and bioimaging, and has demonstrated a time-efficient process for quantitative measurement [14, 15]. Recently, nanostructures have been considered an alternative quenching element as well. For instance, noble plasmonic nanoparticles like gold or silver nanoparticles are often used as fluorescence quencher [16, 17]. Graphene oxide nanosheet (GO) has drawn much attention acting as an emerging fluorescent quencher due to their *π* electrons, single *sp*^2^ carbon layer structure, and the various oxygen-functional groups, such as carboxyl, hydroxyl and epoxy groups [18–20]. FRET quenching system is dependent on the distance between the donor and the quencher. Therefore, when CDs acting as a FRET donor and GO acting as a FRET quencher are in close proximity (< 10 nm), the fluorescence intensity will decrease; and the quenching efficiency highly depends on the distance between the FRET donor and the FRET quencher; when CDs and GO are no longer in close proximity, fluorescence of CDs can be recovered. Such “on” and “off” system can be utilized to study different biomolecules-involved interactions.

In addition, GO has demonstrated superior properties in terms of water solubility, fluorescence quenching ability, strong adsorption via *π*–π* interaction and surface functionalizability [9, 21–23]. GO used in fluorescence sensing system might offer plenty of opportunities for detecting proteins, DNA, molecules, virus, and metal ions [21–25]. Previous studies demonstrate that the adsorption and desorption between short DNA segments and GO through utilizing the electrical properties [26]. The single-strand DNA (ssDNA) can be adsorbed firmly and lie flat on the surface of GO via π–π* interactions; however, the *π*–π* structures can be interrupted by its complementary ssDNA as the two ssDNA hybridize to shield their base pairs with the outer hydrophobic back bone. The newly formed double-strand DNA (dsDNA) is no longer able to be adsorbed onto the surface of GO via non-covalent π-stacking interactions due to its more rigid structure [27, 28]. The process of adsorption and desorption can be used as a biosensing system for detection and quantification.

**Fig. 1:**
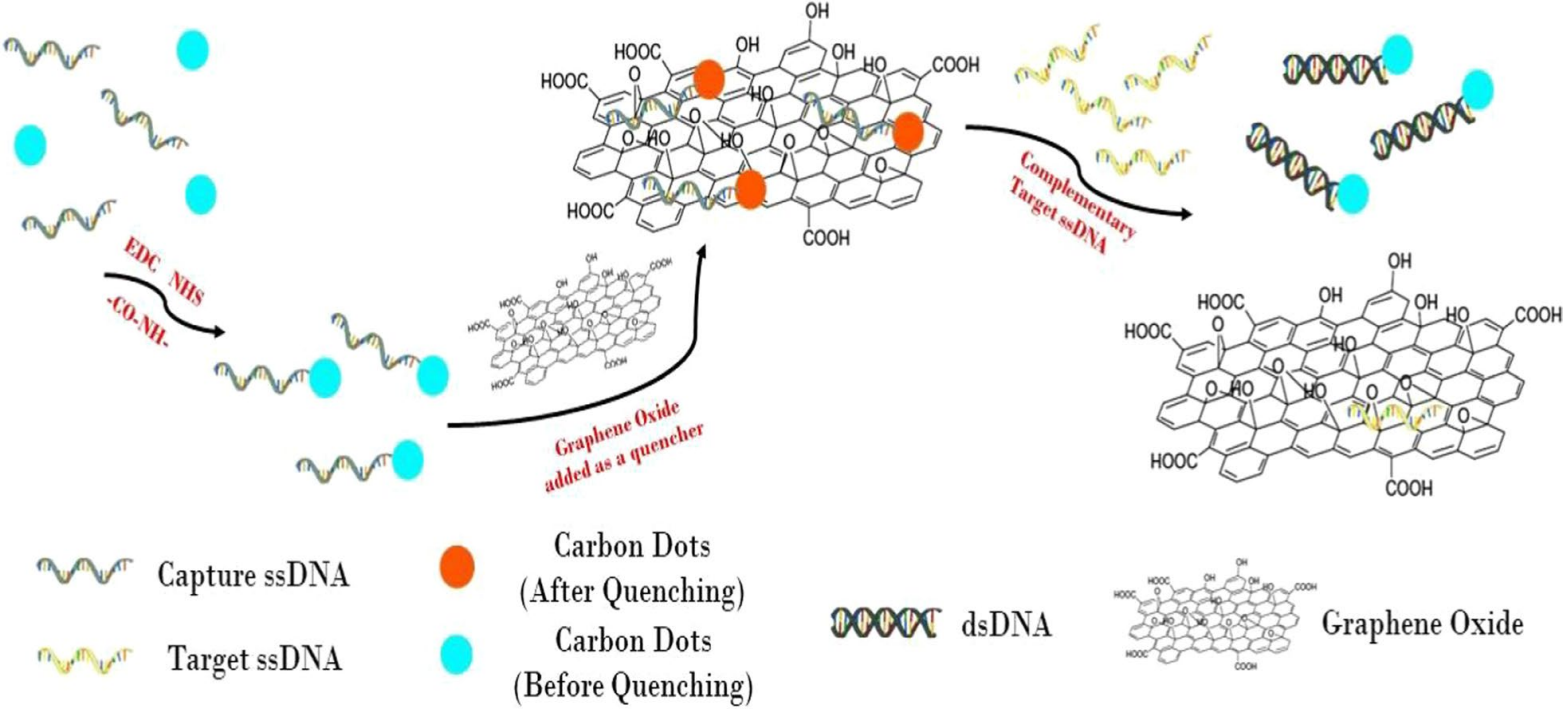
Illustration of the carbon nanostructure-based DNA sensor by using the fluorescence quenching mechanism

In this paper, a new luminescent biosensor by using carbon-based nanomaterials has been developed to detect breast cancer-associated ssDNA in a rapid and accurate manner. As shown in Fig. 1, the biosensor consists of two components: CDs-Capture ssDNA and GO in which CDs acting as FRET donor are bioconjugated with the receptor (i.e., Capture ssDNA). Without Target ssDNA, the two components exist in a complex state where the CDs-Capture ssDNA is bound to GO through *π*–π* interactions. Due to the proximity between CDs and GO, the fluorescence of CDs is quenched. In the presence of Target ssDNA, the hybridization of the two ssDNA is favored, and CDs-Capture ssDNA and GO come apart resulting in the restoration of the fluorescence of CDs. The degree of fluorescence recovery is directly correlated with the concentration of Target ssDNA, and a standard curve can be graphed to illustrate the phenomenon.

## Experimental

### Chemicals and Materials

All the oligonucleotides were purchased from Integrated DNA Technologies. The base sequences used in this study are based on the sequence of Breast Cancer *genes* (BRCA) [29]:19-mer, Target ssDNA: 5′-GAA CAA AAG GAA GAA AAT C-3′. Meanwhile, 25-mer, 5′-GAT TTT CTT CCT TTT GTT CTT TTT T-3′-NH_2_, was chosen as Capture ssDNA. The additional bases—TT TTT T—in the 25 mer works as a flexible linker. Because Target ssDNA is short and relatively rigid. The additional bases of Capture ssDNA could be relatively easier to bond on the surface of CDs; and make the DNA hybridization during the DNA detection more efficiently [28]. On the other hand, other mutant genes were chosen for evaluating the selectivity of the DNA sensor, e.g., 19-mer, 3-base Mismatched ssDNA: 5′-GAA CCA AAT TAA GAA AAT C-3′; 19-mer, Non-base Matched ssDNA: 5′-CTG TTC GCC TGC CGT GGC T-3′. Graphite flake was purchased from Alfa Aesar. N-Hydroxysuccinimide (NHS) was purchased from Fluka. *N*-(3-Dimethylaminopropyl)-*N*′-ethylcarbodiimide hydrochloride (EDC), sulfuric acid, potassium permanganate, hydrochloric acid, citric, and urea were purchased from Sigma-Aldrich.

### Synthesis of CDs

The water-soluble luminescent CDs was prepared by a modified microwave method [30]. 3 g of citric acid and 3 g of urea were dissolved in 10 mL of distilled water, and then microwaved in domestic microwave oven under 750 W for 5 min. After the solution changed from a colorless liquid to a dark-brown clustered solid, the solid was dissolved in distilled water and then transferred to the vacuum oven at 60 °C for around 2 h to remove volatile substance. Finally, CDs were further purified by centrifugation at 3000 rpm for 20 min to remove large water-insoluble particles. CDs powder was obtained by the freeze-dry method.

### Bioconjugation of Capture ssDNA to CDs

The dried CDs powder was resuspended in 1X PBS to achieve a 1 mg/mL concentration. First, 2 mg EDC and 1 mg NHS were added to 100-μL CDs (1 mg/mL) solution then incubated for 2 h at 37 °C to activate carboxyl groups on the surface of CDs. Then, 10-μL amino-modified Capture ssDNA (100 μM) was mixed with 20-μL activated CDs solution at 37 °C. Carboxylic groups on the surface of CDs have been quantified (see the experimental S1 in the Additional file 1). Based on the calculations, around 50% of carboxylic group on the surface of CDs was involved in the bioconjugation. Capture ssDNA was linked to CDs via peptide linkage formed by amino group and carboxyl group. The final products were purified by dialyzing against ddwater with 1 kDa dialysis tube. Finally, the sample of CDs-Capture ssDNA was isolated and stored under 4 °C.

### Development of FRET Quenching System for DNA Detection

GO, the FRET quencher, was prepared by using a modified version of Hummer’s Method [31]. 1 g of graphite flake was added to 50-mL 98% sulfuric acid (H_2_SO_4_) while stirring in an ice-water bath for 5 min. 3 g of potassium permanganate was added, and then stirred for another 25 min at temperature under 10 °C followed 5 min sonication in ultrasonic bath. After repeating the stirring sonication process 12 times, 200 ml of distilled water was added and sonicated for 2 h. Finally, 1 M hydrochloric acid solution (37%) and distilled water were used to wash GO until the pH 6 was reached when centrifuged at 8000 rpm for 30 min. GO powder was isolated by freeze-dry method.

Dry GO powder was resuspended in 1X PBS to achieve a 1 mg/mL concentration. 100 μL of 1 mg/mL GO was used to mix with the 30-μL CDs-Capture ssDNA solution. The mixture was incubated for 15 min to allow for quenching of the fluorescence of the CDs-Capture ssDNA. Finally, the CDs-Capture ssDNA-GO probe was formed, and the quenching system was ready for detection.

### Materials Characterization

UV–Vis spectra of samples were measured by using Agilent Cary 60 UV–Vis. Hydrodynamic diameter and zeta potential of CDs and CDs-Capture ssDNA were measured by Mastersizer (Malvern Instruments Ltd). The microstructures of CDs and GOs were investigated by transmission electron microscope (TEM, FEI Titan 80–300 LB operating at 80 and 300 keV). CDs in aqueous media was dropped on the carbon-coated cu grid for TEM measure. Fourier transform infrared (FTIR, Bruker Vector 22 in the range of 400–4000 cm^−1^) was used to investigate the bioconjugation. The photoluminescence measurements were performed by using fluorophotometry (QuantaMsterTM 30, HORIBA Canada Inc.).

### Evaluation of the Sensing System for Detection of Target ssDNA

CDs-Capture ssDNA-GO quenching system was mixed with Target DNA in different concentrations (from 0.25 to 2.5 µM) at 37 °C for 2 h in dark. The fluorescence signals of the solutions were measured to evaluate the CDs’ fluorescence restoration and recorded under the same condition to make the standard curve of the relationship between the signals change and the concentrations of Target ssDNA. In addition, the selectivity study is conducted by using other two different genes from Target ssDNA, i.e., 3-base mismatched and non-base matched.

## Results and Discussion

### Characterization of the Structures of CDs

**Fig. 2:**
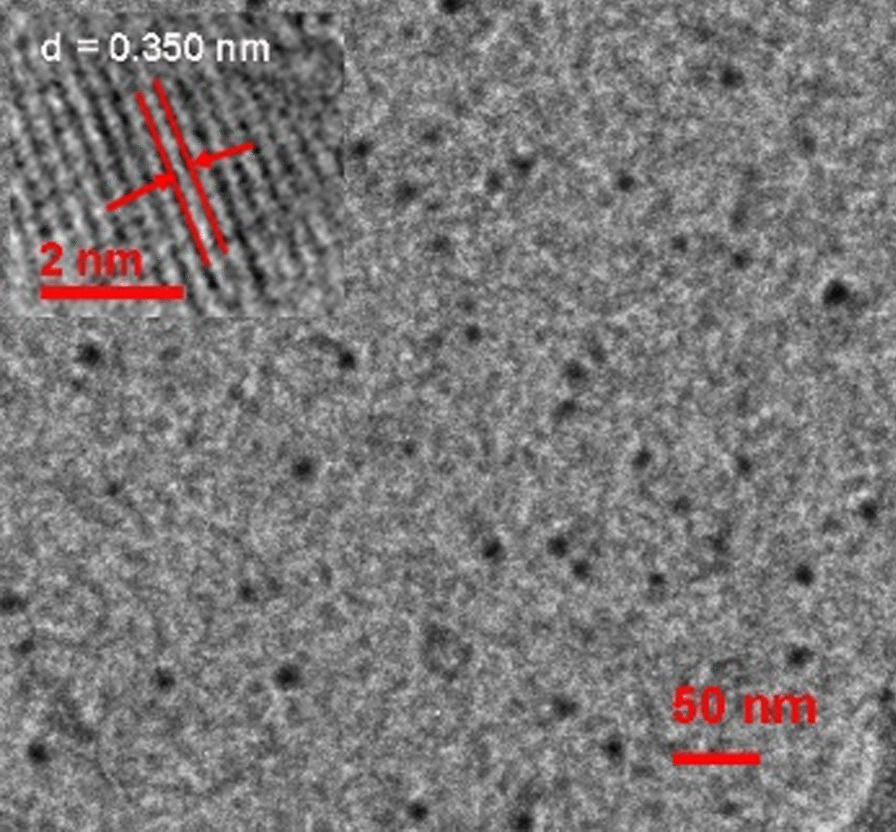
TEM micrograph of CDs, the small inset is the HRTEM micrograph of CDs

TEM and high-resolution TEM (HRTEM) have been employed to study CDs. The average particle size is estimated at 12 ± 3 nm as shown in Fig. 2. The basal plane distance (*d* = 3.50 nm) can be observed in the HRTEM micrograph (the small inset in Fig. 2).

### Investigation of the Bioconjugation of Capture ssDNA to CDs

**Fig. 3:**
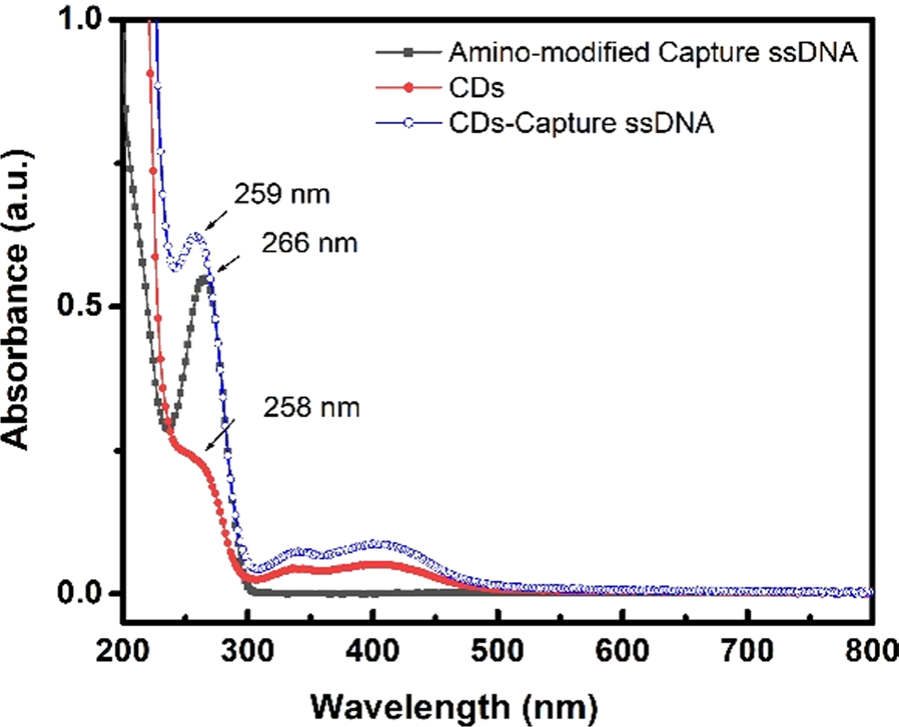
UV–Vis absorbance spectra of amino-modified Capture ssDNA, CDs, and the CDs-Capture ssDNA

The absorbance of samples, i.e., amino-modified Capture ssDNA, CDs, and the CDs-Capture ssDNA, have been measured by UV–Vis spectrometer as shown in Fig. 3. The amino-modified Capture ssDNA has a typical absorbance peak at 266 nm. CDs show three absorption peaks in UV–Vis spectrum centering at 258 nm, 336 nm and 400 nm. The multi-absorption of CDs is caused by the multifunctional groups and the nitrogen-doping into CDs [32]. CDs-Capture ssDNA also shows three absorption peaks in its UV–Vis spectrum, one of them at 256 nm with 10 nm of left-shift to the absorbance of the free amino-modified Capture ssDNA and another two peaks attributed from CDs without obvious shift. The results of UV–Vis absorbance indicate that the amino-modified Capture ssDNA has been bioconjugated to CDs successfully.

Zeta potential (*ξ*) of CDs and CDs-Capture ssDNA is -9.53 mV and − 4.24 mV, respectively. The negative value of ξ to CDs results from the functional groups on the surface of CDs, e.g., carboxy group. The increased ξ after conjugating Capture DNA on CDs is caused by the reaction between carboxyl group on the surface of CDs and 3′ amino group from Capture ssDNA.

**Fig. 4:**
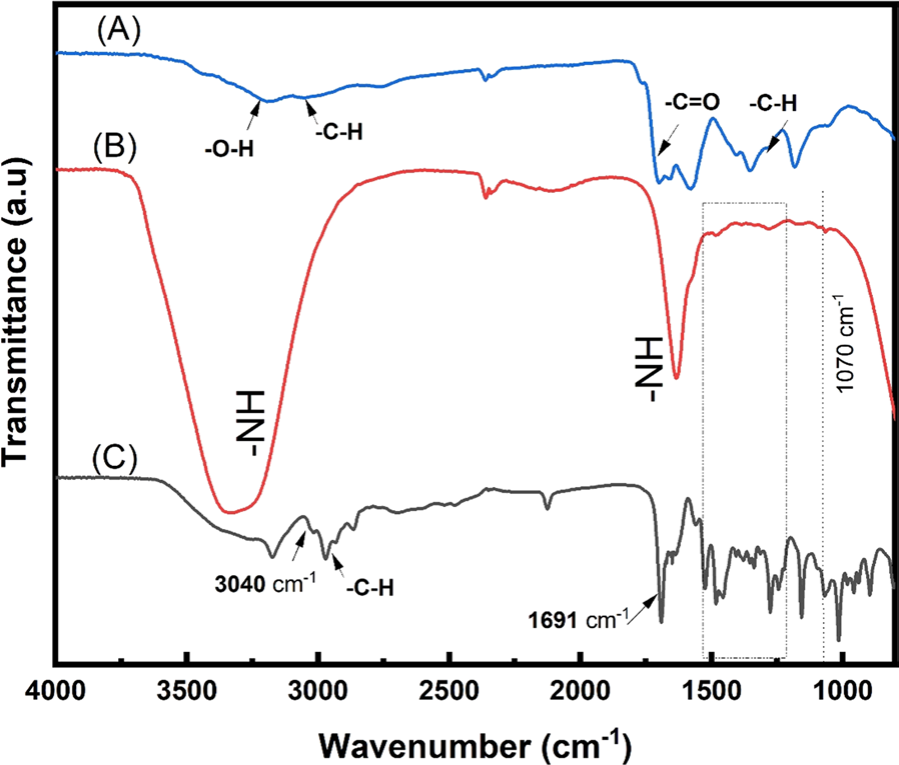
FTIR spectra of **A** CDs, **B** amino-modified Capture ssDNA, and **C** CDs-Capture ssDNA

To further investigation of the bioconjugation of Capture ssDNA onto CDs, FTIR has been used in measuring different samples. The FTIR spectra of CDs, amino-modified Capture ssDNA and CDs-Capture ssDNA are shown in Fig. 4. The peaks at 1700 cm^−1^ (Fig. 4A) is attributed to the stretch of –C=O bond in the carboxylic group on the surface of CDs [33]. The peaks at 1353 cm^−1^, 1579 cm^−1^ in the FTIR spectrum of CDs (Fig. 4A) are attribute to C–O–H bending and C=C stretching, respectively. The peaks around 3174 cm^−1^ and 1633 cm^−1^ shown in Fig. 4B is attributed to N–H bending, which demonstrates the amino group of Capture ssDNA. The amide band at1690 cm^−1^ and 3040 cm^−1^ in Fig. 4B, corresponding to –C=O stretching(amide bond) and N–H vibration (amide I band), demonstrates that the formation of peptide bond (–CO–NH–) [34]. The result indicates the successful bioconjugation of Capture ssDNA to CDs. Peaks at 2971 cm^−1^ and 1484 cm^−1^ in the FTIR spectra of CDs (Fig. 4A) and CDs-Capture ssDNA (Fig. 4C) indicate the presence of C–H stretching. In addition, the DNA base–sugar vibration in the range of 1260 cm^−1^ to 1500 cm^−1^ and the peak of phosphate group at 1066 cm^−1^ can be observed in both of Fig. 4B, C, which indicates that the DNA structure remains after the conjugation process.

### Photoluminescence of CDs With and Without Bioconjugation of Capture ssDNA

**Fig. 5:**
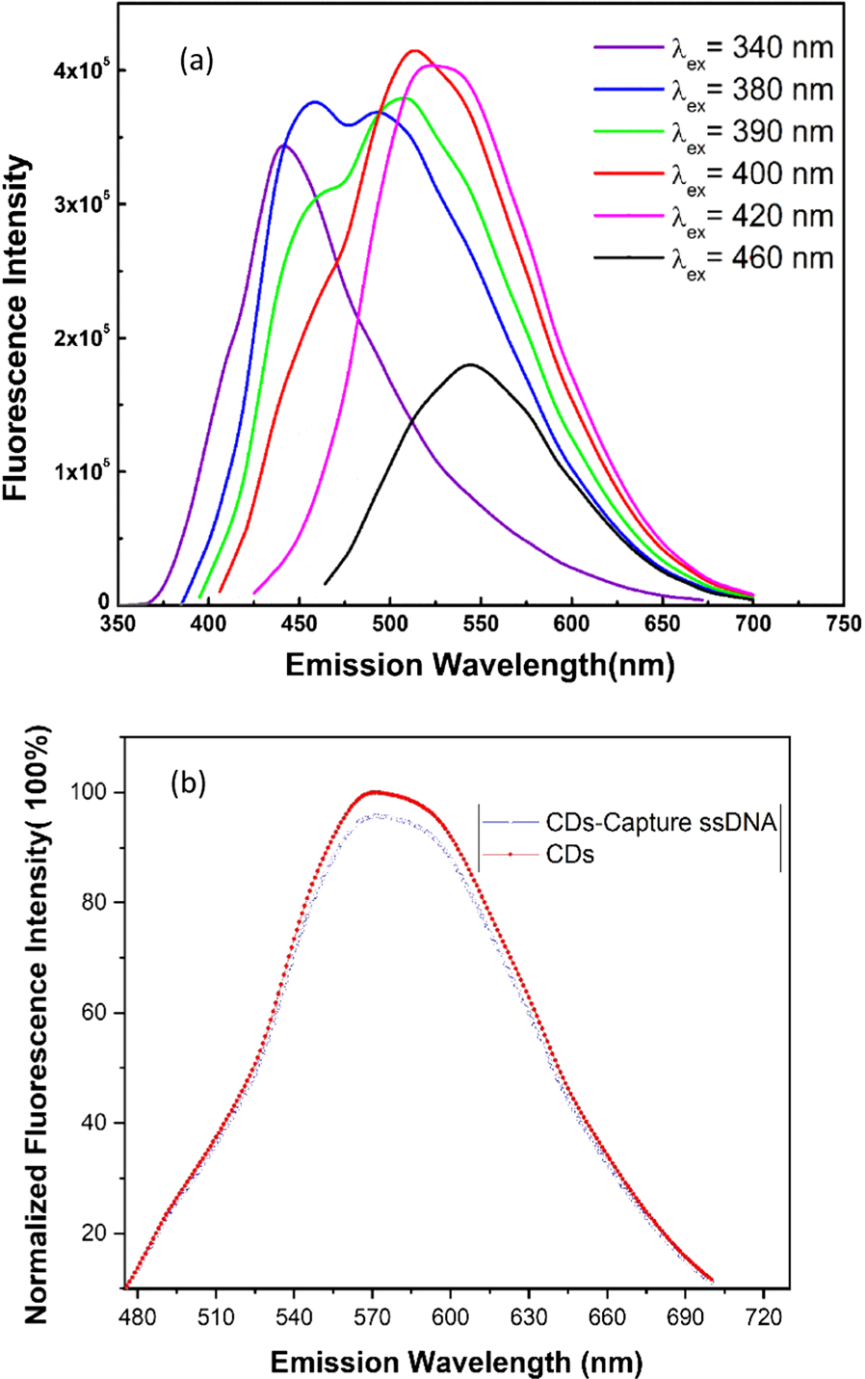
**a** Photoluminescence (PL) of CDs under different excitation wavelengths. **b** PL measure of CDs and CDs-Capture ssDNA, both has the same concentration of CDs

The fluorescence emission (*λ*_em_) intensity of CDs has been measured under different excitation wavelength (*λ*_ex_). Figure 5 shows that *λ*_em_ of CDs shifts to right from 440 to 545 nm when *λ*_ex_ increases from 340 to 460 nm; and the maximum fluorescence intensity (*I*_max_) is observed at *λ*_em_ = 510 nm when *λ*_ex_ = 400 nm as shown in Fig. 5a. Therefore, all photoluminescence (PL) of CDs-based samples had been measure under *λ*_ex_ = 400 nm. The PL of CDs before and after bioconjugating with Capture ssDNA has been measured. It is found that the maximum fluorescence intensity (*I*_max_) of both samples, i.e., CDs and CDs-Capture ssDNA, can be observed at *λ*_em_ = 510 nm, whereas *I*_max_ of CDs-Capture ssDNA decreases 5% compared to that of CDs. It could be caused by the change of surface states of CDs after bioconjugating with ssDNA (Fig. 5b). Meanwhile, the PL spectra of CDs physically mixing with ssDNA were measured. The *I*_max_ of CDs remains the same before and after physically mixing with ssDNA (Additional file 1: Fig. S3). This result further confirms the bioconjugation of Capture ssDNA to CDs.

### Characterization of the Fluorescence Quencher

**Fig. 6:**
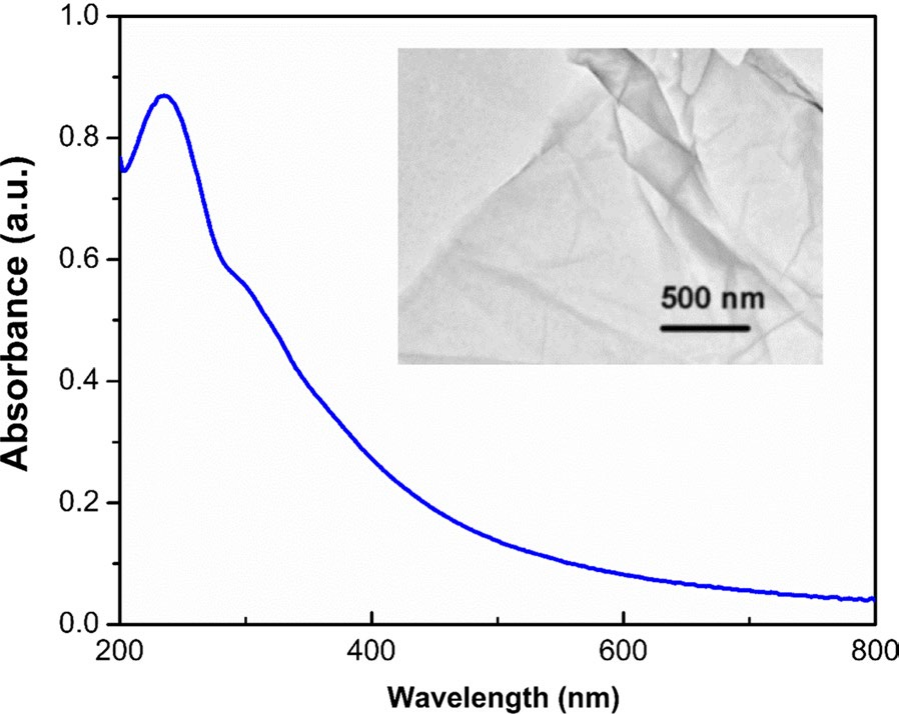
UV–Vis absorbance of graphene oxide (GO); the small inset is the TEM micrograph of GO

Two-dimensional carbon-based nanostructure materials, i.e., GO, has been considered a strong fluorescence quencher due to its wide absorption in the ultraviolet range. Figure 6 displays the absorption of GO by using UV–vis spectrophotometer; the typical absorption peak of the GO at 236 nm is observed which corresponds to the *π* → π^*^ transition of the aromatic *π* electrons [35]. A small absorption peak at 303 nm is related to *n* → π^*^ transition of the C=O bonds on GO [35]. The TEM micrograph (inset image) indicates the single layer of GO.

### Performance of the Carbon-Based DNA Sensing System

**Fig. 7:**
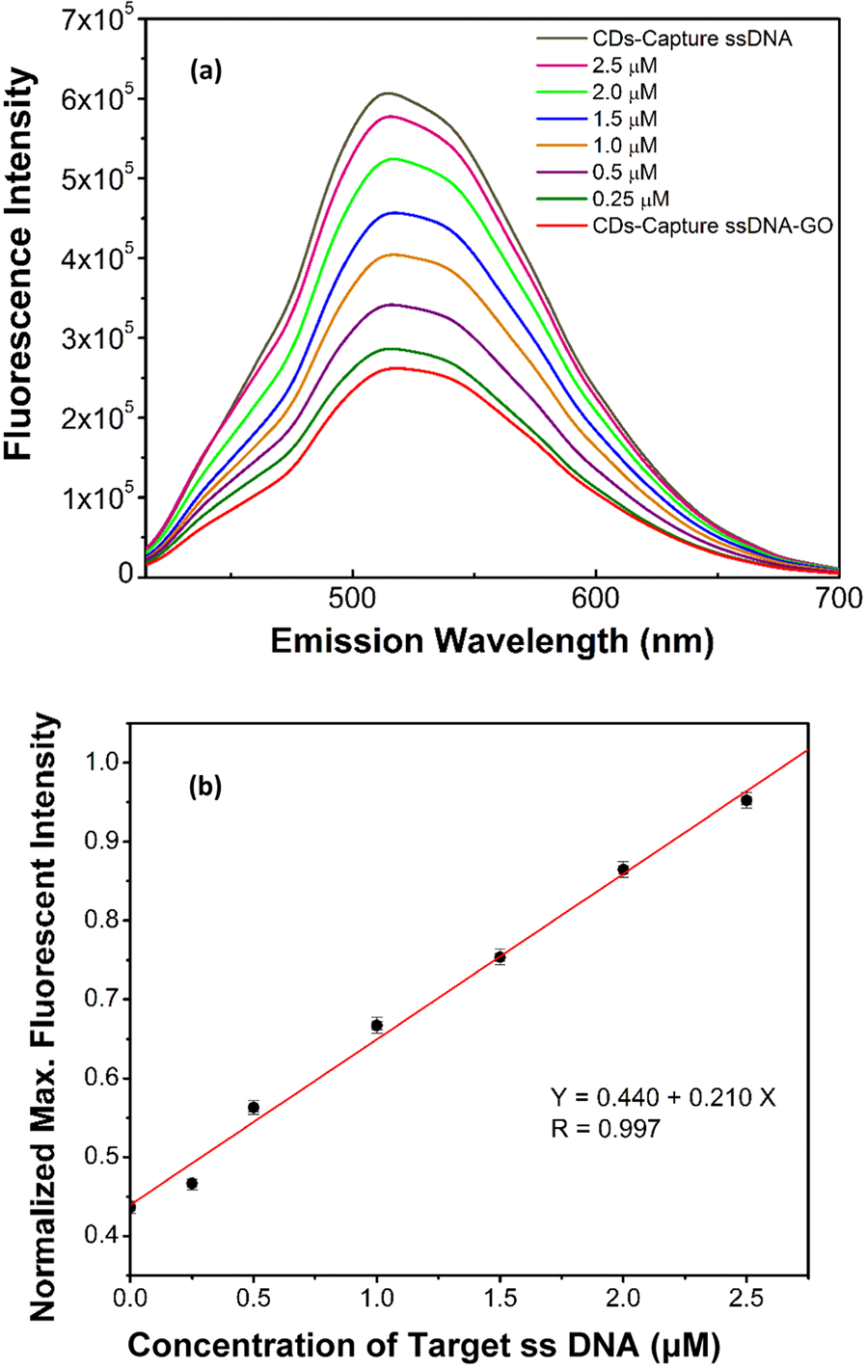
Photoluminescence (PL) properties of the carbon nanostructure-based DNA sensor used to detect target ssDNA. **a** PL of CD-Capture ssDNA and CD-Capture ssDNA-GO under *λ*_ex_ = 400 nm; and the restoration of PL of CD-Capture ssDNA-GO when it is introduced the complementary Target ssDNA with different concentrations. **b** A linear relationship between the normalized maximum fluorescence intensity (*I*_max_) and the concentration of complementary Target ssDNA

The optimal ratios of CDs to Capture ssDNA, and CDs-Capture ssDNA to GO, respectively were studied (see Additional file 1: Figure S2, Figs. 5, 7a). To develop a homogeneous DNA sensor with optimal ratios, 20 μL 1 mg/mL CDs, 10 μL 100 μM capture ssDNA, and 100 uL 1 mg/mL GO were applied in building the FRET quenching system. Then, 50 uL of the quenching system, i.e., CDs-Capture ssDNA-GO, were used for detecting different amount of target ssDNA. The PL properties of the CDs-based DNA sensing system were measured before/after interacting with GO. *I*_max_ of CDs-Capture ssDNA decreases over 57.4% after interacting with GO. When Target ssDNA is present in the sensing system made of CD-Capture ssDNA-GO, the reduced *I*_max_ restores, and normalized *I*_max_ increases from 42.6% to 95.2% with an increase in the concentration of Target ssDNA from 0 to 2.5 μM under the excitation wavelength at 400 nm (shown in Fig. 7a). A linear relationship *y* = 0.440 + 0.210*x* is obtained by normalizing the maximum fluorescence intensity (*I*_max_) and the concentration of Target ssDNA as shown in Fig. 7b. The detection limit is around 0.24 μM. On the other hand, different controls, CDs, CDs bioconjugated with Capture ssDNA (CDs-Capture ssDNA), CDs physically mixed with Capture ssDNA and GOs and CDs mixed with GOs have been further studied to find out the PL properties of the different systems. The quenching and restoring phenomena of the FRET-based sensing system in detecting target ssDNA are not observed (Additional file 1: Figs. S4–S8).

**Fig. 8:**
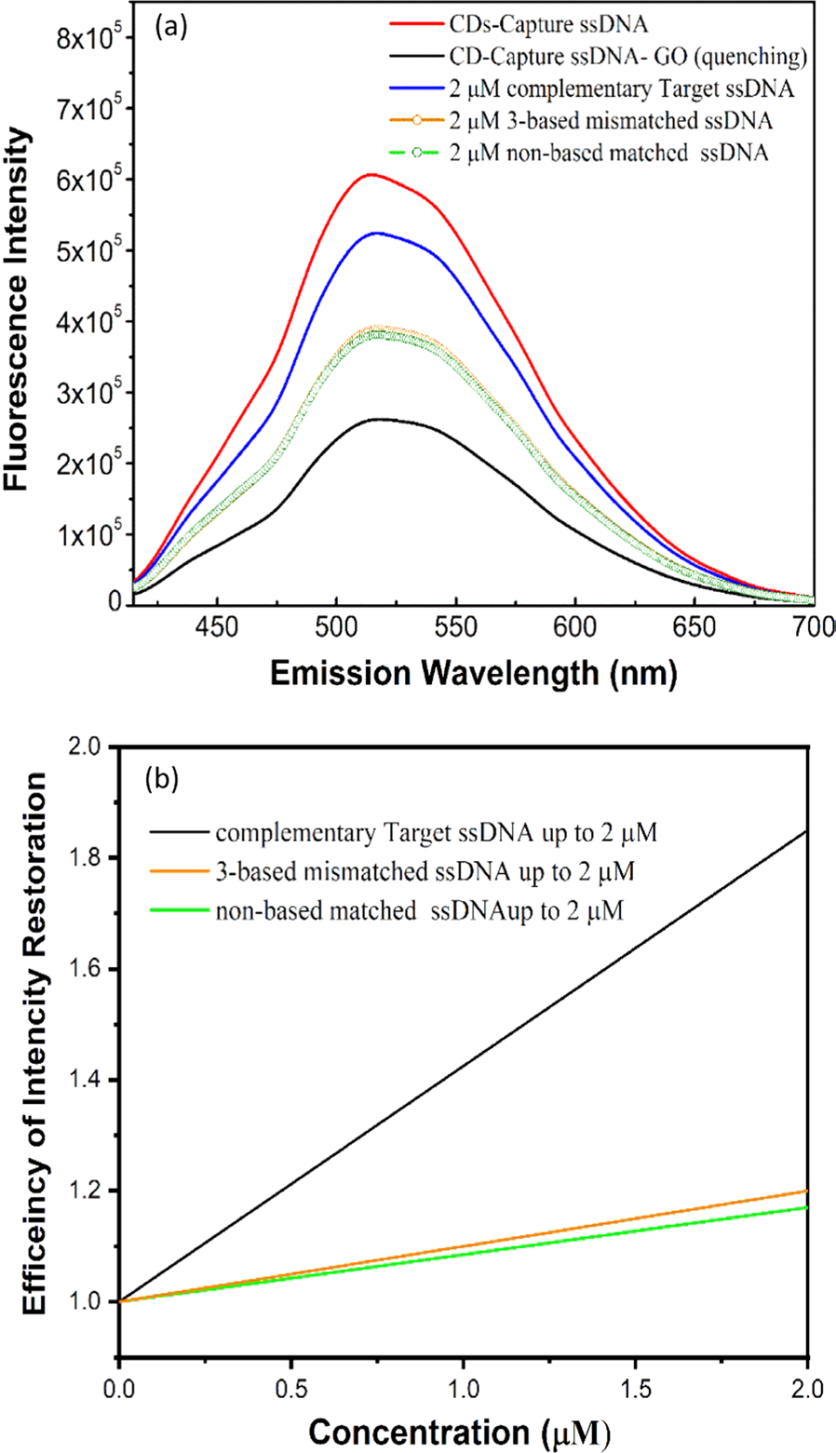
Selectivity study by detecting the mismatch sequences with the same concentration of complementary Target ssDNA. **a** PL of different samples. **b** linear relationship of intensity as a function of concentration of different samples

On the other hand, mismatch testing has been investigated to evaluate the selectivity of the carbon nanostructure-based DNA sensor. Different ssDNA (complementary Target ssDNA, 3-base mismatched ssDNA and non-base matched ssDNA) with the same concentration (2 μM) were measured by using the carbon-based nanostructured-DNA sensing system. As shown in Fig. 8a, the *I*_max_ of the carbon-based nanostructured-DNA sensor system is restored to 1.89 times of times of the *I*_max_ of CD-Capture ssDNA-GO compared to quenching system, approaching to 85% of the *I*_max_ of CDs-Capture ssDNA, when the complementary Target ssDNA with 2 μM presents in the system, whereas the *I*_max_ of the sensing system is restored inefficiently for measuring the same concentration of 3-base mismatched ssDNA and non-base matched ssDNA; the normalized *I*_max_ is 1.17 times of the *I*_max_ of CD-Capture ssDNA-GO. It is noted that the major difference in selectivity test among targeted ssDNA, 3-base mismatched ssDNA, non-base matched ssDNA is related to the slope of the linear function as shown in Fig. 8b. Thus, the method to determine the size of a DNA fragment, e.g., electrophoresis, should be associated with the analysis of selectivity.

## Conclusions

In summary, a carbon nanostructure-based DNA sensor has been developed which composes of CDs-Capture ssDNA and GO where CDs acts as a donor of FRET quenching system and GO, the fluorescence quencher. The average diameter of CDs is at 12 ± 3 nm. The bioconjugation of Capture ssDNA onto CDs has been investigated and verified through the measurements by using UV–Vis spectrophotometer, FTIR and fluorospectrometer. Due to π–π interaction between the CDs-Capture ssDNA and GO, the FRET quenching occurs; the *I*_max_ of CDs-Capture ssDNA decreases over 57.4% after interacting with GO. In the presence of complementary Target ssDNA, the FRET quenching between CDs and GO was disrupted due to the association constant between target ssDNA and CDs-Capture ssDNA through hydrogen bond is bigger than that between GO and CDs-Capture ssDNA. Therefore, the PL of CDs-Capture ssDNA restores, and *I*_max_ at *λ*_em_ = 510 nm increases with increasing concentration of Target ssDNA from 0.25 to 2.5 μM. A linear relationship of *I*_max_ and Target ssDNA concentration is observed. The detection limit is around 0.24 μM. In addition, the results of the mismatch test show good sensitivity of the carbon nanostructure-based sensor. Although this sensor system is made to detect ssDNA associate with breast cancer, the sensor can be easily modified to have a different set of Capture and Target ssDNA, which showcases its limitless potential in diagnosing different types of diseases. Furthermore, due to the unique fluorescence properties of CDs and wide range absorption properties of GO, fluorescence signals can be measured at different excitation wavelengths if that is more appropriate for the biological setting.

## Supplementary Information

**Additional file 1: Experiment-S1.** Quantification of Carboxylic Groups on the Surface of CDs. **Experiment-S2.** Photoluminescence of CDs physically mixed with ssDNA sequences. **Experiment-S3.** Control studies.

## Data Availability

All the data and material are available in the manuscript.

## Abbreviations

CDs: Carbon quantum dots
GO: Graphene oxide nanosheet
ssDNA: Single-strand DNA
dsDNA: Double-strand DNA
TEM: Transmission electron microscopy
HRTEM: High-resolution transmission electron microscopy
FRET: Förster resonance energy transfer
NHS: N-hydroxysuccinimide
EDC: *N*-(3-Dimethylaminopropyl)-*N*′-ethylcarbodiimide hydrochloride
FTIR: Fourier-transform infrared spectroscopy
UV–Vis: Ultraviolet–visible spectroscopy
PL: Photoluminescence
