# Supplementary material for: Carbon Nanostructure-Based DNA Sensor Used for Quickly Detecting Breast Cancer-Associated Genes
Source: Nanoscale Res Lett. 2022 Sep 20;17:93. doi: 10.1186/s11671-022-03730-3 (PMC9489825; doi:10.1186/s11671-022-03730-3)
Supplement: Supplementary file 1 — Additional file 1: Experiment-S1. Quantification of Carboxylic Groups on the Surface of CDs. Experiment-S2. Photoluminescence of CDs physically mixed with ssDNA sequences. Experiment-S3. Control studies. [file 11671_2022_3730_MOESM1_ESM.docx]

**Supplementary Materials**

Carbon Nanostructure-based DNA Sensor Used for Quickly Detecting

Breast Cancer-associated Genes

Yingqi Zhang ^a^, Jisu Song ^b^, Songlin Yang ^a^, Jianying Ouyang ^c^, and Jin Zhang ^a,b*^

^a^ Chemical and Biochemical Engineering, University of Western Ontario, London, Ontario, Canada, N6A 5B9
^b^ School of Biomedical Engineering, University of Western Ontario, London, Ontario, Canada, N6A 5B9

^c^ National Research Council Canada, 1200 Montreal Road, Ottawa, ON K1A 0R6, Canada.

**Experiment-S1. Quantification of Carboxylic Groups on the Surface of CDs**

Acid-base titration was involved for the quantification of carboxylic groups on the surface of CDs. 2 mL CDs (1 mg/mL) were mixed well with 2 mL NaOH (0.01 M) solution for 2 hrs under stirring. After achieving the equilibrium (where the pH was stable) between the CDs and NaOH by converting all carboxylic groups to their sodium salt (equation (1)), HCl (0.01 M) solution was applied to titrate the excess NaOH (equation (2)). To reach the end point of the titration, around 1980 uL HCl solution was consumed which indicated that 20 uL 0.01 M NaOH were involved in the first reaction. Therefore, 0.2 μmol carboxylic groups were involved in the first reaction where the ratio could be determined around 0.2 μmol carboxylic groups/ 2 mg CDs.

-COOH + NaOH → -COONa + H_2_O equation (1)

HCl + NaOH → NaCl + H_2_O equation (2)


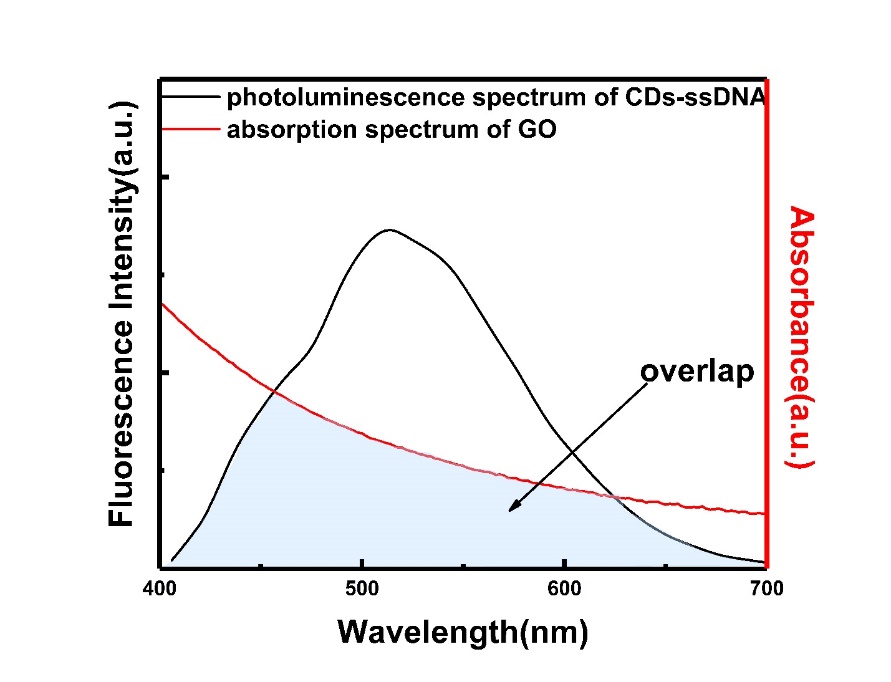


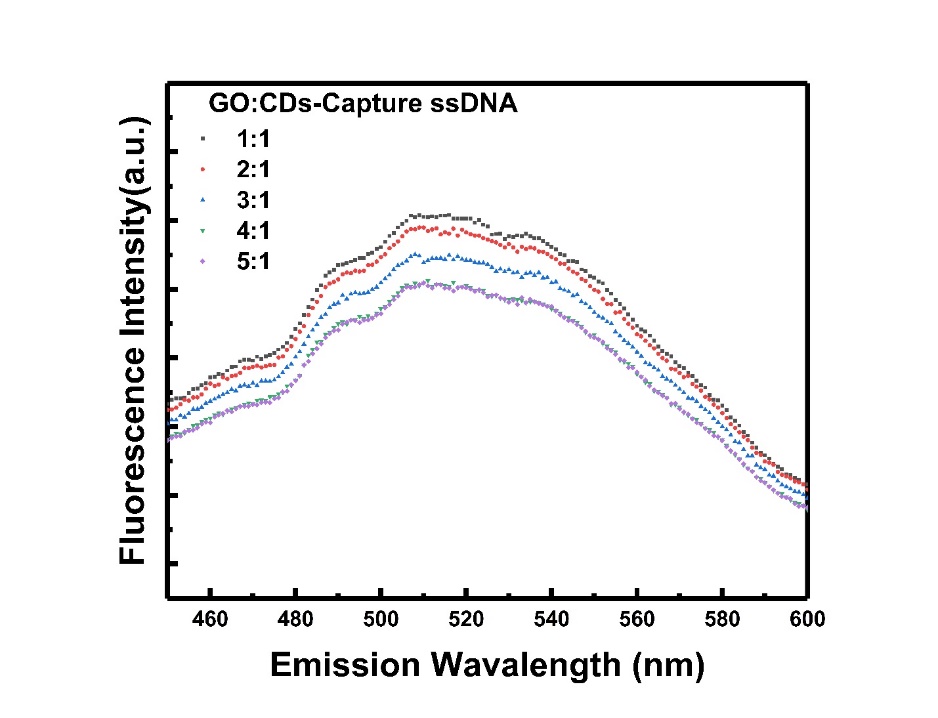
**Fig. S1.** Photoluminescence (PL) of CDs-Capture ssDNA under excitation wavelength at λ_ex_ = 400 nm measured by fluorospectrometer; and the UV absorbance of GO measured by UV-vis spectrometer.

**Fig. S2.** Photoluminescence (PL) of FRET quenching system with the ratio of CDs-Capture ssDNA to GO under excitation wavelength at λ_ex_ = 400 nm.


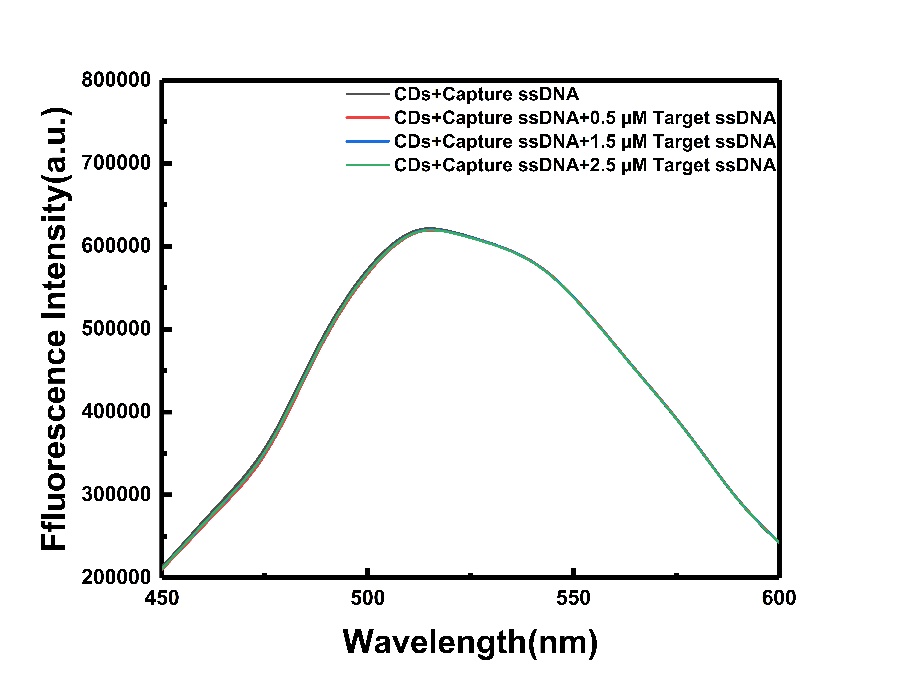
**Experiment-S2. Photoluminescence of CDs physically mixed with ssDNA sequences**

**Fig. S3**. Photoluminescence (PL) properties of CDs physically mixed with capture ssDNA and then mixed with Complementary target ssDNA in different concentrations.

**Experiment-S3. Control studies**

*S3-1. Fluorescence properties of CDs mixing with Complementary target ssDNA.*


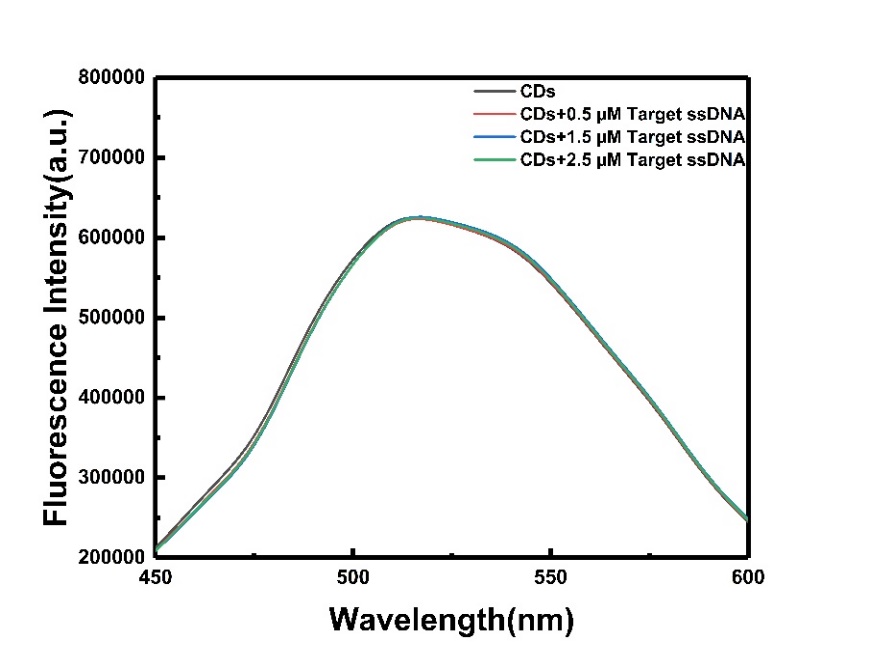


**Fig. S4**. Photoluminescence (PL) properties of CDs mixed with Complementary target ssDNA in different concentrations.


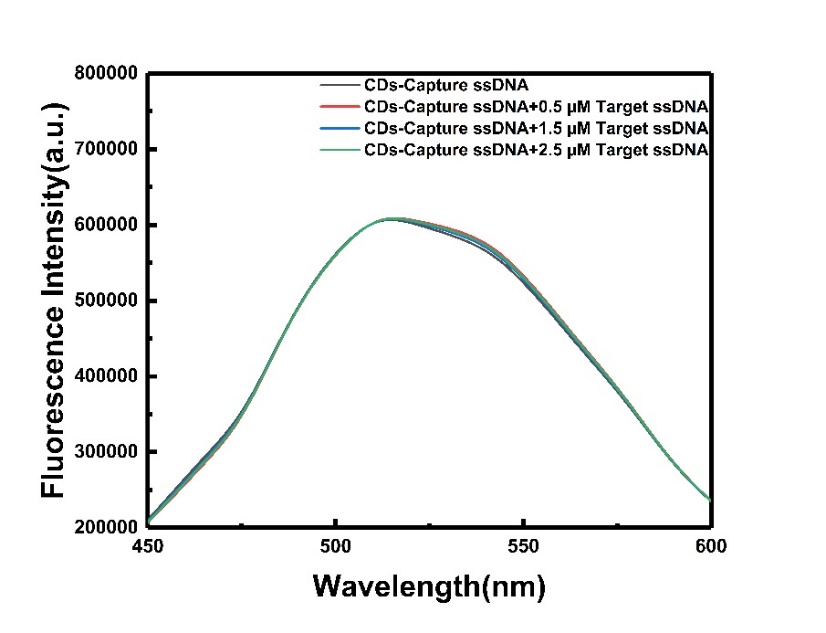
*S3-2. Fluorescence properties of CDs-Capture ssDNA mixing with Complementary target ssDNA.*

**Fig. S5**. Photoluminescence (PL) properties of CDs bioconjugated with capture ssDNA (CDs-Capture ssDNA), and the PL of CDs-Capture ssDNA mixed with Complementary target ssDNA in different concentrations.


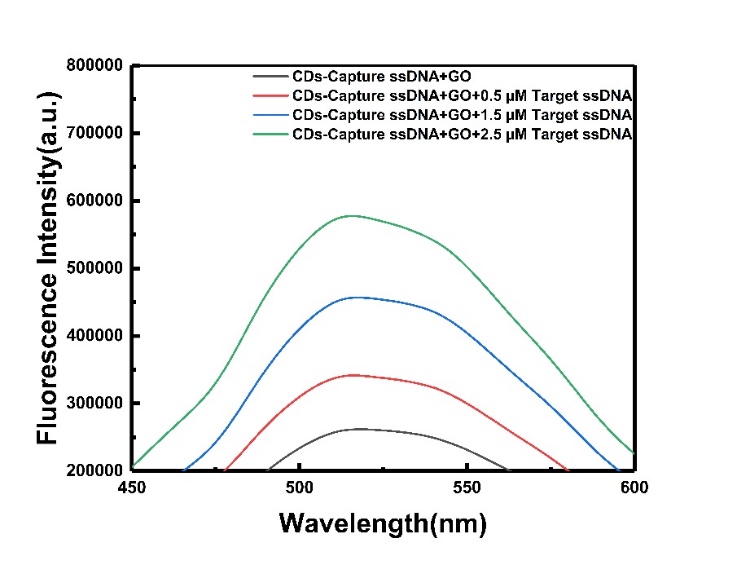
*S3-3. Fluorescence properties of the FRET sensing system made of CDs-Capture ssDNA-GOs, and the PL of sensing system corresponding to the presence of complementary target ssDNA.*

**Fig. S6.** The photoluminescence (PL) properties of the carbon nanostructure-based DNA sensor used to detect target ssDNA. Here, CDs conjugated with capture ssDNA which quenched by GO and then mixed with Complementary target ssDNA in different concentrations, The result is compatible with Fig. 7a


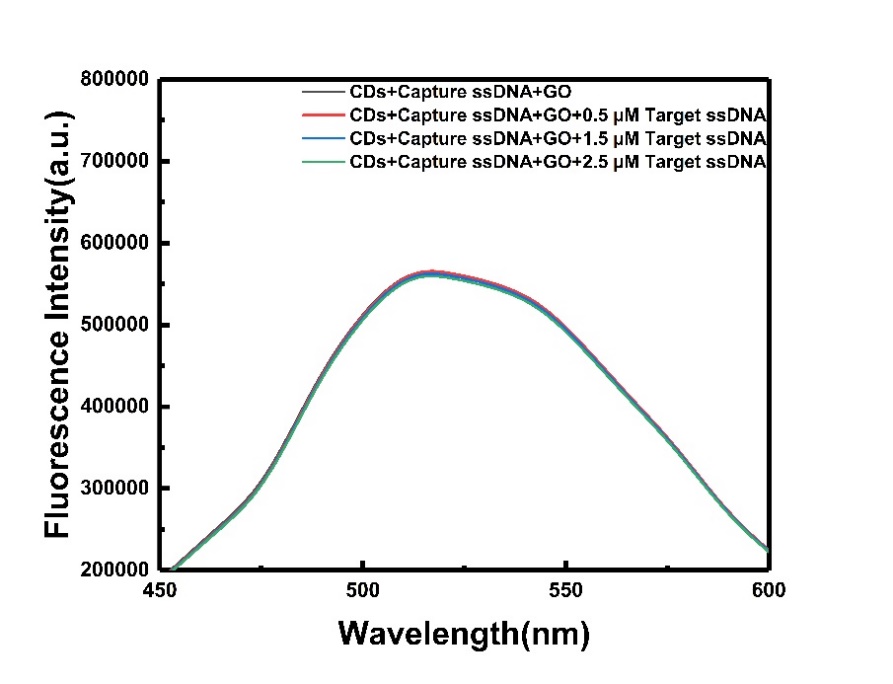
*S3-4. Fluorescence properties of CDs physically mixed with Capture ssDNA-GOs and GOs (CDs+Capture ssDNA + GOs), and the PL corresponding to the presence of complementary target ssDNA.*

**Fig. S7**. The photoluminescence (PL) properties *of CDs physically mixed with Capture ssDNA-GOs and GOs (CDs+Capture ssDNA + GOs), and the PL corresponding to the presence of complementary target ssDNA.*


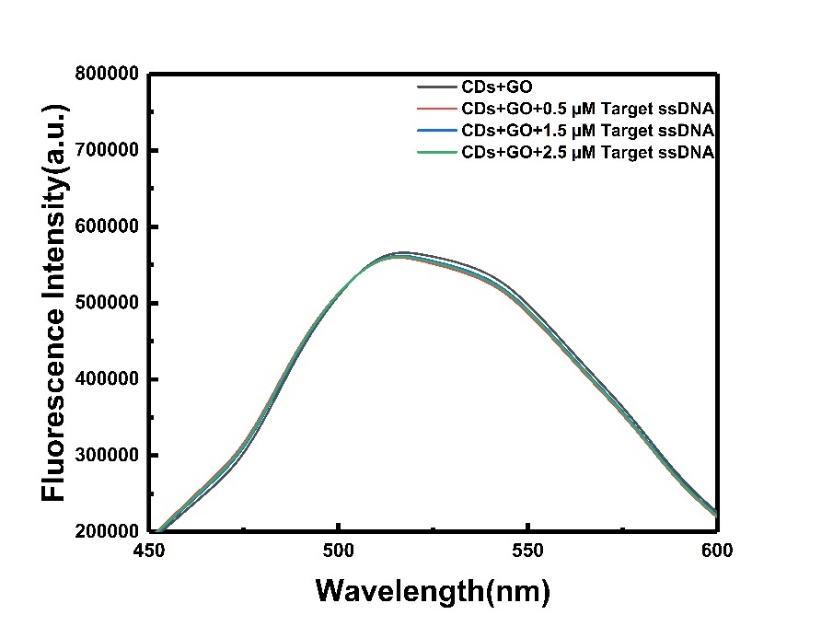
*S3-5. Fluorescence properties of CDs physically mixed with GOs, and the PL of CDs corresponding to the presence of complementary target ssDNA.*

**Fig. S8**. The photoluminescence (PL) properties *of* CDs mixed with GO and then mixed with Complementary target ssDNA in different concentrations.
